# Retinography as a marker of the progression of cognitive impairment and/or dementia in older people with type II diabetes mellitus

**DOI:** 10.3389/fnagi.2026.1909175

**Published:** 2026-10-30

**Authors:** Francisco Javier Sánchez-Jiménez, Vanesa Cantón-Habas, María José Reyes-Medina, María del Pilar Carrera-González

**Affiliations:** 1 Department of Nursing, Pharmacology and Physiotherapy, Faculty of Medicine, Nursing and Physiotherapy, University of Córdoba, Córdoba, Spain; 2 Instituto Maimónides de Investigación Biomédica de Córdoba (IMIBIC), Córdoba, Spain; 3 Hospital Universitario Reina Sofía, Córdoba, Spain; 4 Experimental and Clinical Physiopathology Research Group CTS-1039, Department of Health Sciences, Faculty of Health Sciences, University of Jaén, Jaén, Spain

**Keywords:** cognitive impairment, dementia, type 2 diabetes mellitus, aged, retinography

## Abstract

**Introduction:**

The aging population increases type 2 diabetes mellitus (T2DM) prevalence and consequently heightens related complications, including dementia and microvascular disease. This study examined the relationship between diabetic retinopathy (DR) and cognitive decline in adults with T2DM.

**Methods:**

A prospective cohort study was conducted with 328 individuals aged ≥60 years with T2DM in Córdoba, Spain. Cognitive function was assessed using the Mini-Mental State Examination (MMSE) at baseline, 6, and 12 months, and retinal imaging identified DR. Clinical and sociodemographic data were collected.

**Results:**

MMSE scores declined from 28.31 (SD = 2.07) at baseline to 27.47 (SD = 2.43) at 12 months (*p* < 0.001). At 12 months, participants with DR exhibited significantly lower MMSE scores (26.75 ± 2.93 vs. 27.58 ± 2.27; *p* = 0.03). Multimorbidity and hypertension were associated with accelerated cognitive decline.

**Discussion:**

DR appears as a marker of microvascular-related cognitive decline in T2DM, highlighting the value of combined ophthalmologic and cognitive monitoring.

## 1 Introduction

Population aging represents one of the major health challenges of the 21st century and is closely linked to an increasing burden of chronic non-communicable diseases, such as type 2 diabetes mellitus (T2DM) (Hambleton et al., 2023; The Lancet, 2023; Ong et al., 2023). Indeed, the prevalence of T2DM has risen steadily in recent decades, with current estimates indicating that between 7 and 8% of the adult population in Europe is affected, increasing to approximately 25% among individuals aged ≥65 years (Ong et al., 2023; Ogurtsova et al., 2022). This trend is driven not only by population aging but also by the marked rise in risk factors accompanied by a higher incidence of both microvascular and macrovascular complications (Ong et al., 2023; Lu et al., 2023; American Diabetes Association Professional Practice Committee et al., 2024).

Diabetic retinopathy stands out among chronic complications associated with T2DM due to its prevalence, affecting approximately 22% of patients, and its potential value as a clinical marker of systemic damage. Its onset and progression are closely associated with disease duration, poor glycemic control, and other metabolic comorbidities (Lu et al., 2023; Bianco et al., 2022; Lin et al., 2021).

In this regard, the increasingly robust scientific evidence linking T2DM to a heightened risk of neurodegenerative diseases, particularly Alzheimer’s disease (AD), the most common form of dementia, followed by vascular dementia, has gained considerable relevance. Risk estimates suggest that individuals with diabetes are 1.5–2.5 times more likely to develop these conditions than their non-diabetic counterparts (Beeri and Bendlin, 2020). The pathophysiological substrate of T2DM and dementia shares several mechanisms, supporting the hypothesis of a pathological continuum between these entities (Beeri and Bendlin, 2020; Biessels et al., 2020; Kellar and Craft, 2020; Srikanth et al., 2020; Launer, 2020; Van Sloten et al., 2020; Wu et al., 2025).

Moreover, due to its neuroectodermal origin and microvascular characteristics that closely resemble those of the brain, the retina is increasingly proposed as a key structure for the early assessment of cognitive decline in individuals with T2DM (Beeri and Bendlin, 2020; Biessels et al., 2020). Recent studies suggest that retinal findings may mirror central neurodegenerative processes, opening new avenues for the use of ocular imaging technologies as non-invasive diagnostic tools (Van Sloten et al., 2020).

Despite the clear pathophysiological and epidemiological association, the specific molecular and biological mechanisms linking neurodegenerative diseases and T2DM remain insufficiently elucidated. In this sense, some studies indicate a stronger association with cerebrovascular damage than with hallmark AD lesions, such as amyloid plaques or neurofibrillary tangles (Aderinto et al., 2023), which do not exclude a potential link between AD and vascular dementia (Brenowitz et al., 2017).

It also remains unclear whether there is a direct and/or temporal causal relationship or if both share common risk factors without synergistic pathogenic interaction. Likewise, randomized clinical trials have not provided consistent evidence that intensive glucose control lowers dementia risk in individuals with diabetes, although lifelong diabetes- free status is associated with lower dementia risk (Cao et al., 2024). Altogether, these issues underscore the need for new therapeutic strategies and a deeper understanding of the underlying processes (Zhao et al., 2025).

Essential questions, such as whether retinopathy functions as a causal marker or merely reflects the degree of systemic microvascular involvement, or whether different subtypes and severity levels convey distinct predictive value depending on the type of dementia remain to be clarified (Van Sloten et al., 2020; Sartore et al., 2023). In this context, quantitative retinal vascular biomarkers, including reduced fractal dimension, increased vessel tortuosity, and alterations in retinal vessel caliber, have emerged as potential indicators of cerebral microvascular dysfunction, reflecting vascular rarefaction, endothelial injury, blood–retinal barrier impairment, and cerebral hypoperfusion, all of which are closely linked to central neurodegenerative processes (Van Sloten et al., 2020). Similarly, the sensitivity and specificity of retinal changes for detecting subclinical cognitive impairment in relation to dementia onset must be better established, as most available studies are observational and clinical trials are lacking to determine whether early detection and management of retinopathy could influence cognitive outcomes (Van Sloten et al., 2020).

The final step should be to establish standardized protocols integrating ophthalmological assessment into routine screening for cognitive impairment in older adults with T2DM, if its diagnostic or prognostic value is confirmed.

Therefore, the objective of this study is to analyze the association between diabetic retinopathy and cognitive impairment in a cohort of individuals over 60 years of age diagnosed with T2DM, analyzing/evaluating its potential as an early biomarker of dementia. To this end, we aim to determine the prevalence of diabetic retinopathy in older people with T2DM, its distribution according to severity, and its evolution after 1 year of monitoring their cognitive status, establishing an intermediate point at 6 months. The possible involvement of clinical and sociodemographic factors will also be studied.

## 2 Methods

### 2.1 Study design

A prospective cohort study was conducted in primary healthcare centers within the Northern Health District of Córdoba, Spain, between January 2024 and July 2025. The follow-up included three visits: a baseline assessment and two follow-up visits at 6 and 12 months (Figure 1).

**Figure 1:**
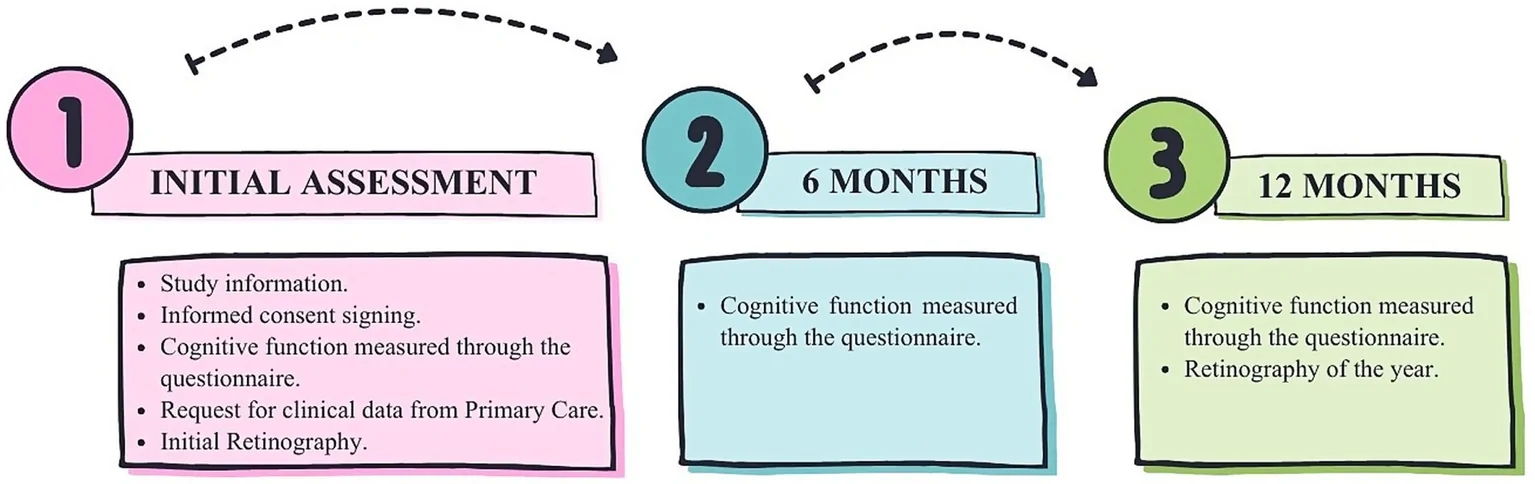
Timeline of data collection points.

### 2.2 Participants and selection criteria

A total of 328 participants were enrolled through simple random sampling among individuals scheduled for retinal imaging (retinography) within the regional Diabetes Care Programme. This program is part of an integrated diabetes care pathway that ensures coordinated follow-up between primary and hospital care, and includes routine diabetic retinopathy screening using digital fundus photography from the time of diabetes diagnosis. More specifically, this examination is carried out by nursing staff every 1 or 2 years in people without diabetic retinopathy, and annually or more frequently in those with retinal lesions or other high-risk factors. In this sense, eligible individuals were selected from the program’s appointment registry, ensuring equal probability of selection for all candidates meeting the initial screening requirements. The study specifically focused on older adults with T2DM; therefore, an age threshold of ≥60 years was established because this population represents a clinically relevant stage at which diabetes-related microvascular damage and early cognitive changes become increasingly prevalent, while many individuals remain free of clinically overt dementia. This provides an appropriate clinical window to evaluate diabetic retinopathy as a potential early biomarker of cognitive impairment.

Inclusion criteria were:

- A confirmed diagnosis of T2DM.
- Age ≥ 60 years at the time of recruitment.
- Provision of written informed consent, either directly by the participant or via an authorized family member or legal representative when required.

Exclusion criteria included:

- A documented diagnosis of major neurocognitive disorder (dementia) at baseline, identified through clinical records or initial cognitive screening.
- Presence of an acute psychiatric disorder that hindered completion of the cognitive or complementary assessments.
- People diagnosed with Down’s syndrome, due to their specific neurodevelopmental profile and the significantly higher risk of developing AD and early-onset dementia, which could constitute a potential confounding factor in the assessment of diabetes-related cognitive decline.

The sample size was calculated based on a reference population comprising older adults from various municipalities within the Northern Health District of Córdoba, with an average population of 5,316 individuals. The required sample size was estimated using EPIDAT 4.2. Assuming an expected prevalence of approximately 30% for T2DM, a confidence level of 95%, and a precision of 5%, the sample size was determined to be 305 older adults with T2DM.

### 2.3 Study measures

#### 2.3.1 Dependent variables

The primary dependent variables were cognitive function, assessed using the Lobo Mini-Mental State Examination (MMSE), administered at baseline, 6 months, and 12 months following the retinal imaging (retinography) test, and the presence of retinopathy detected during the initial retinography assessment. Retinal imaging was performed as part of the regional Diabetes Care Programme using a Topcon TRC-NW400 non-mydriatic retinal camera (Topcon Corporation, Tokyo, Japan) with a 45° field of view. Images were acquired under standardized conditions and stored in JPEG format with a resolution of 1,956 × 1,934 pixels in RGB color space. Before image acquisition, image quality was assessed, and pharmacological mydriasis with cyclopentolate hydrochloride was performed when image quality was considered inadequate. Images were considered acceptable when they demonstrated adequate focus, sufficient illumination and contrast, and were free of motion artifacts or media opacities that could compromise the identification of retinal lesions. In this sense, images not meeting these quality criteria were repeated whenever possible or excluded from the analysis. Three color fundus photographs were obtained from each eye, corresponding to the macular, central, and optic disc fields. Images from both eyes were subsequently uploaded to the retinal screening platform of the Andalusian Public Health System for specialist assessment within Primary Care. Retinal images were graded according to the International Clinical Diabetic Retinopathy Disease Severity Scale (ICDR), which is routinely implemented within the regional diabetic retinopathy screening program. The platform records eye laterality (right or left), duration of diabetes, and whether pupillary dilation was performed for each examination. The presence of diabetic retinopathy was determined according to the specialist ophthalmological assessment recorded in the screening program.

#### 2.3.2 Independent variables

Independent variables included sociodemographic characteristics (sex, age, marital status, education level); laboratory parameters obtained within 3 months prior to the retinography, including fasting plasma glucose, LDL cholesterol, HDL cholesterol, total cholesterol, glycated hemoglobin (HbA1c), and hemoglobin concentration; level of dependence in performing basic activities of daily living (ADLs), measured using the Barthel Index; and clinical history including medical antecedents, diagnoses, pharmacological treatments, and the presence of diabetes-related complications (nephropathy, diabetic foot, neuropathy, microvascular and macrovascular involvement).

### 2.4 Statistical analysis

Univariate and bivariate were performed. Specifically, for descriptive analysis, qualitative variables were expressed as absolute frequencies and percentages, while quantitative variables were summarized using means and standard deviations. For bivariate analysis, the Student’s *t*-test was used to compare means between two independent groups after confirming normality of the data distribution. When normality assumptions were not met, the non-parametric Mann–Whitney U test was applied. Additionally, for comparisons among more than two independent groups, one-way analysis of variance (ANOVA) was conducted. When parametric assumptions were violated, the non-parametric Kruskal–Wallis test was used. Additionally, longitudinal changes in MMSE scores were assessed using a linear mixed-effects model. Time was included as a fixed effect and participant as a random intercept to account for repeated measurements within individuals. An unstructured covariance matrix was specified, and Bonferroni-adjusted pairwise comparisons of estimated marginal means were performed. A *p*-value of <0.05 was considered statistically significant for all analyses. Statistical analyses were performed using SPSS software, version 29.

### 2.5 Ethical aspects

The study was conducted in accordance with the ethical principles outlined in the Belmont Report and the Declaration of Helsinki. Additionally, current data protection legislation was strictly observed, specifically the Spanish Organic Law 3/2018 of December 5th on Personal Data Protection and Guarantee of Digital Rights. This study received favorable ethical approval from the Andalusian Research Ethics Committee, under reference number 5648 of session No. 350 held on June 28, 2023.

## 3 Results

### 3.1 Sample characteristics

A total of 328 participants were included in the study, of whom 71.06% were women. The overall mean age of the sample was 69.98 years (SD = 6.45; range 60–86). Statistically significant age differences were observed by sex (*p* = 0.01), with women showing a higher mean age compared to men (71.06 ± 6.33 vs. 69.24 ± 6.45 years, respectively).

Significant sex differences were observed in marital status (*p* < 0.001) and educational level (*p* = 0.002). Widowhood and lower educational attainment were more prevalent among women, whereas men presented higher rates of marriage and university education. No differences were found in socioeconomic level (*p* = 0.42).

Clinically, the most prevalent comorbidity was hypertension (59.15%), followed by dyslipidemia (22.87%) and depression (11.28%). The prevalence of depression was significantly higher among women than men (20.45% vs. 5.10%; *p* < 0.001), whereas dyslipidemia was more frequent in men (27.04%) than women (16.67%; *p* = 0.03). No significant differences were observed between sexes in hypertension (*p* = 0.83), previous retinopathy (*p* = 0.32), cardiovascular risk (*p* = 0.91), or multimorbidity (*p* = 0.61). The mean duration of diabetes was 11.66 years (SD = 7.03), with no differences between women and men (11.51 ± 7.23 vs. 11.75 ± 6.91 years; *p* = 0.77). Regarding diabetes-related characteristics, the mean fasting plasma glucose was 133.09 ± 40.30 mg/dL and the mean HbA1c was 7.03 ± 1.14%. Overall, 21.65% of participants were receiving insulin therapy, whereas 94.21% were treated with oral glucose-lowering medications. Table 1 summarizes the sociodemographic and clinical characteristics of the study population by sex. In addition, baseline characteristics according to diabetic retinopathy status are summarized in Table 2. Compared with participants without diabetic retinopathy, those with diabetic retinopathy showed significantly higher fasting plasma glucose levels (144.64 ± 51.47 vs. 131.33 ± 37.82 mg/dL, *p* = 0.03), higher HbA1c values (7.57 ± 1.51 vs. 6.94 ± 1.02%, *p* < 0.001), and a higher prevalence of insulin therapy (37.04% vs. 18.61%, *p* = 0.002). The two groups were otherwise comparable with respect to sociodemographic characteristics, diabetes duration, oral glucose-lowering medication use, lipid profile, hemoglobin concentration, and the remaining baseline clinical variables.

**Table 1:** Description of the sample according to sex.

	Total *n* = 328	Women *n* = 132	Men *n* = 196	*p*-value
Age (years) x̅ (SD)	69.98 (6.45)	71.06 (6.33)	69.24 (6.45)	0.01
Marital status *n* (%)				
Single	20 (6.12)	1 (0.76)	19 (9.69)	<0.001
Married	228 (69.72)	75 (57.25)	153 (78.06)	
Widowed	56 (17.13)	49 (37.40)	7 (3.57)	
Divorced	23 (7.03)	6 (4.58)	17 (8.67)	
Level of education *n* (%)				
Primary education	263 (80.43)	118 (90.08)	145 (73.98)	0.002
Basic education/bechalor	23 (7.03)	7 (5.34)	16 (8.16)	
Professional training	29 (8.87)	4 (3.05)	25 (12.66)	
University degree	12 (3.67)	2 (1.53)	10 (5.10)	
Economic level *n* (%)				
Low	150 (45.70)	58 (43.94)	92 (46.94)	0.42
Medium	176 (53.66)	74 (56.06)	102 (52.04)	
High	2 (0.61)	–	2 (1.02)	
Barthel index x̅ (SD)	97.27 (6.91)	95.57 (5.04)	98.42 (8.75)	0.002
Clinical variables *n* (%)				
Depression	37 (11.28)	27 (20.45)	10 (5.10)	< 0.001
Dyslipidaemia	75 (22.87)	22 (16.67)	53 (27.04)	0.03
Hypertension	194 (59.15)	79 (59.85)	115 (58.67)	0.83
Pretinal retinopathy	52(17.81)	24 (20.51)	28 (16)	0.32
Cardiovascular risk	116 (36.02)	46 (35.66)	70 (36.20)	0.91
Pluripathology	92 (28.05)	35 (26.52)	57 (29.08)	0.61
Diabetes-related characteristics				
Diabetes diagnosis time (years) x̅ (SD)	11.66 (7.03)	11.51 (7.23)	11.75 (6.91)	0.77
Fasting plasma glucose (mg/dL) x̅ (SD)	133.09 (40.3)	130.23 (42.63)	135.03 (38.63)	0.3
Glycated hemoglobin (%) x̅ (SD)	7.03 (1.14)	7.03 (1.12)	7.03 (1.16)	0.97
Oral antidiabetics *n* (%)	309 (94.21)	124 (93.94)	185 (94.39)	0.86
Insulin therapy *n* (%)	71 (21.65)	34 (25.76)	37 (18.88)	0.14
Diabetic nephropathy *n* (%)	101 (30.89)	34 (25.76)	67 (34.36)	0.09
Diabetic foot *n* (%)	5 (1.53)	–	5 (2.56)	–
Diabetic neuropathy *n* (%)	6 (1.83)	4 (3.03)	2 (1.03)	–
Microvascular involvement *n* (%)	39 (11.93)	8 (6.06)	31 (15.9)	0.007
Macrovascular involvement *n* (%)	80 (24.46)	29 (21.97)	51 (26.15)	0.39
Laboratory parameters				
LDL colesterol (mg/dL) x̅ (SD)	84.98 (29.83)	90.68 (28.63)	81.06 (30.09)	0.007
HDL colesterol (mg/dL) x̅ (SD)	47.95 (14.22)	53.05 (12.44)	44.5 (14.35)	<0.001
Total colesterol (mg/dL) x̅ (SD)	161.44 (35.87)	171 (33.44)	154.98 (36.1)	<0.001
Hemoglobin (g/dL) x̅ (SD)	14.31 (1.79)	13.59 (1.41)	14.81 (1.86)	<0.001

**Table 2:** Sociodemographic characteristics according to the presence or absence of diabetic retinopathy.

	Retinopathy	Non retinopathy	*p*-value
Age (years) x̅ (SD)	69.41 (7.22)	70.09 (6.3)	0.15
Sex *n* (%)			
Men	37 (68.52)	159 (58.03)	0.16
Women	17 (31.48)	115 (41.97)	
Marital status *n* (%)			
Single	8 (14.81)	12 (4.40)	0.04
Married	35 (64.81)	193 (70.70)	
Widowed	8 (14.81)	48 (17.58)	
Divorced	3 (5.56)	20 (7.33)	
Level of education *n* (%)			
Primary education	43 (79.63)	220 (80.59)	0.87
Basic education /Bechalor	4 (7.41)	19 (6.96)	
Professional training	4 (7.41)	25 (9.16)	
University degree	3 (5.56)	9 (3.30)	
Economic level *n* (%)			
Low	25 (46.30)	125 (45.62)	0.45
Medium	28 (51.85)	148 (54.01)	
High	1 (1.85)	1 (1.36)	
Clinical variables *n* (%)			
Depression *n* (%)			
Yes	3 (5.56)	34 (12.41)	0.14
No	51 (94.44)	240 (87.59)	
Dyslipidaemia *n* (%)			
Yes	11 (20.37)	64 (23.36)	0.55
No	43 (79.63)	210 (76.64)	
Hypertension *n* (%)			
Yes	29 (53.70)	165 (60.22)	0.36
No	25 (46.30)	109 (39.78)	
Pretinal retinopathy *n* (%)			
Yes	27 (51.92)	25 (10.42)	<0.001
No	25 (48.08)	215 (89.58)	
Cardiovascular risk *n* (%)			
Yes	19 (35.19)	97 (36.19)	0.88
No	35 (64.81)	171 (63.81)	
Pluripathology *n* (%)			
Yes	20 (37.04)	72 (26.28)	0.13
No	34 (62.96)	202 (73.72)	
Diabetes-related characteristics			
Diabetes diagnosis time (years) x̅ (SD)	13.12 (7.13)	12.2 (6.56)	0.38
Fasting plasma glucose (mg/dL) x̅ (SD)	144.64 (51.47)	131.33 (37.82)	0.03
Glycated hemoglobin (%) x̅ (SD)	7.57 (1.51)	6.94 (1.02)	<0.001
Oral antidiabetics *n* (%)	51 (94.44)	258 (94.16)	0.94
Insulin therapy *n* (%)	20 (37.04)	51 (18.61)	0.002
Diabetic nephropathy *n* (%)	20 (37.74)	81 (29.56)	0.26
Diabetic foot *n* (%)	1 (1.89)	4 (1.46)	–
Diabetic neuropathy *n* (%)	1 (1.89)	5 (1.82)	–
Microvascular involvement *n* (%)	6 (11.32)	33 (12.04)	0.82
Macrovascular involvement *n* (%)	14 (26.42)	66 (24.09)	0.82
Laboratory parameters			
LDL colesterol (mg/dL) x̅ (SD)	90.1 (36.29)	83.96 (28.34)	0.17
HDL colesterol (mg/dL) x̅ (SD)	44.9 (11.74)	48.56 (14.61)	0.1
Total colesterol (mg/dL) x̅ (SD)	162.72 (41.07)	161.19 (34.84)	0.74
Hemoglobin (g/dL) x̅ (SD)	14.78 (1.57)	14.22 (1.82)	0.05

### 3.2 Cognitive status progression

The mean Mini-Mental State Examination (MMSE) score decreased over the 12-month follow-up (baseline: 28.31 ± 2.07; 6 months: 28.01 ± 2.20; 12 months: 27.47 ± 2.43). Statistically significant declines were observed between baseline and 6 months (*p* = 0.0002), baseline and 12 months (*p* < 0.0001), and between 6 and 12 months (*p* < 0.0001), indicating a progressive deterioration of cognitive function over time.

To further evaluate these longitudinal changes while accounting for the correlation between repeated measurements within participants, a linear mixed-effects model was performed. The analysis demonstrated a significant overall effect of time on MMSE scores [*F*(2, 307.05) = 43.29, *p* < 0.001]. The estimated marginal means (EMMs; SE) were 28.31 (0.11) at baseline, 27.96 (0.13) at 6 months, and 27.45 (0.14) at 12 months. Pairwise comparisons of the EMMs, adjusted using the Bonferroni correction, demonstrated significant differences between baseline and 6 months (mean difference = 0.35, 95% CI 0.14–0.56; *p* < 0.001), baseline and 12 months (mean difference = 0.86, 95% CI 0.64–1.09; *p* < 0.001), and between 6 and 12 months (mean difference = 0.51, 95% CI 0.31–0.71; *p* < 0.001).

### 3.3 Relationship between diabetic retinopathy and cognitive function

At baseline, MMSE scores did not differ significantly between participants with and without diabetic retinopathy (27.93 ± 2.83 vs. 27.58 ± 2.27; *p* = 0.15). Although lower MMSE scores were observed among participants with diabetic retinopathy at 12 months (26.75 ± 2.93 vs. 27.58 ± 2.27; *p* = 0.03), these unadjusted comparisons were further examined using the adjusted linear mixed-effects model.

The adjusted longitudinal linear mixed-effects model demonstrated that diabetic retinopathy was independently associated with lower MMSE scores after controlling for age, sex, educational level, and diabetes duration [type III test: *F*(1, 295.98) = 7.06, *p* = 0.008]. Participants without diabetic retinopathy scored, on average, 0.89 MMSE points higher than those with diabetic retinopathy (β = 0.888, SE = 0.360, 95% CI 0.18–1.60, *t* = 2.47, *p* = 0.014). No significant interaction was observed between time and diabetic retinopathy [type III test: *F*(2, 286.22) = 1.87, *p* = 0.157], indicating that the association between diabetic retinopathy and MMSE scores remained stable throughout the 12-month follow-up.

### 3.4 Relationship between clinical factors and cognitive function

Multimorbidity was consistently associated with lower MMSE scores at baseline (27.82 ± 2.24 vs. 28.50 ± 1.97; *p* = 0.007), 6 months (27.43 ± 2.27 vs. 28.23 ± 2.13; *p* = 0.004), and 12 months (26.53 ± 2.66 vs. 27.82 ± 2.24; *p* < 0.0001). Hypertension was significantly associated with lower MMSE scores only at 12 months (27.18 ± 2.63 vs. 27.87 ± 2.04; *p* = 0.01). No significant associations were observed for depression, dyslipidemia, prior retinopathy, or cardiovascular risk at any time point. Detailed results are presented in Table 3.

**Table 3:** Association between clinical variables and cognitive function (MMSE) at baseline, 6 months, and 12 months.

	Clinical variables								
	Basaline x̅ (SD)		*p*-value	6 months x̅ (SD)		*p-*value	12 months x̅ (SD)		*p*-value
Depression	Yes	28.41 (1.71)	0.76	Yes	27.97 (1.80)	0.91	Yes	27.40 (1.88)	0.08
	No	28.29 (2.11)		No	28.01 (22.24)		No	27.48 (2.49)	
Dyslipidaemia	Yes	28.55 (1.98)	0.26	Yes	28.18 (2.14)	0.45	Yes	27.55 (2.30)	0.74
	No	28.24 (2.09)		No	27.96 (2.21)		No	27.44 (2.47)	
Hypertension	Yes	28.16 (2.1)	0.13	Yes	27.82 (2.31)	0.07	Yes	27.18 (2.63)	0.01
	No	28.51 (2.01)		No	28.27 (1.99)		No	27.87 (2.04)	
Pre-existing retinopathy	Yes	28.23 (2.04)	0.84	Yes	27.60 (2.04)	0.22	Yes	27.38 (2.56)	0.81
	No	28.30 (2.1)		No	28.02 (2.24)		No	27.38 (2.56)	
Cardiovascular risk	Yes	28.14 (1.97)	0.25	Yes	27.75 (2.22)	0.17	Yes	27.15 (2.25)	0.12
	No	28.41 (2.11)		No	28.11 (2.19)		No	27.60 (2.52)	
Pluripathology	Yes	27.82 (2.24)	0.007	Yes	27.43 (2.27)	0.004	Yes	26.53 (2.66)	<0.001
	No	28.50 (1.97)		No	28.23 (2.13)		No	27.82 (2.24)	

The duration of diabetes was significantly associated with MMSE scores at baseline, 6 months, and 12 months. At baseline, longer diabetes duration predicted lower MMSE scores (*p* < 0.001), with the regression equation:

MMSE_baseline_ = 28.85–0.0452 × years with diabetes (*R*^2^ = 2.41%).

At 6 months, the association remained significant (*p* = 0.001):

MMSE_6 months_ = 28.66–0.05 × years with diabetes (*R*^2^ = 2.98%).

At 12 months, a significant relationship was also observed (*p* = 0.005):

MMSE_12 months_ = 28.13–0.06 × years with diabetes (*R*^2^ = 2.71%).

These findings indicate that longer diabetes duration is associated with a small but statistically significant decline in cognitive performance over 1 year, although the proportion of variance explained is modest.

## 4 Discussion

The results of this study confirm that DR may serve as a significant peripheral marker of cognitive decline in patients with T2DM, suggesting that the microvascular and neurodegenerative processes associated with diabetes extend beyond ocular involvement (Simó-Servat et al., 2019). Although the presence of this diabetic complication at baseline assessment did not show a significant association with cognitive performance, the 12-month follow-up data reveal a more pronounced decline in cognitive scores among patients with DR. This finding, consistent with the hypothesis that DR reflects subclinical neurovascular damage and may constitute an early marker of neurological vulnerability, suggests a cumulative effect of microvascular dysfunction on brain function. These results align with previous studies linking diabetic microangiopathy to mild cognitive impairment, dementia, and deficits in executive function and memory (Madonna et al., 2017; Nian et al., 2021; Mrugacz et al., 2021).

From a physiological perspective, the retina and brain tissue share key structural and functional characteristics, including a highly specialized microvasculature and homologous endothelial barriers, the blood-retinal and blood–brain barriers (Mrugacz et al., 2021; Huang, 2020; Mills et al., 2021). Pathologically, the sustained hyperglycemia characteristic of T2DM induces the formation of advanced glycation end-products (AGEs), oxidative stress, and low-grade systemic inflammation, promoting endothelial dysfunction, cerebral hypoperfusion, and progressive neuronal loss (Yonamine et al., 2023; Wang et al., 2021; Li et al., 2023). These processes manifest in the brain as structural alterations in critical regions for cognition, such as the hippocampus and prefrontal cortex, where structural atrophy and reduced functional connectivity have been observed, explaining the cognitive decline seen in our cohort in domains reliant on vascular and neuronal integrity (Yonamine et al., 2023; Martínez-Martos et al., 2024). Thus, diabetic retinopathy (DR) emerges as an accessible biomarker that allows inference of the extent of cerebral microvascular damage at early stages, prior to the appearance of overt clinical symptoms (Zhang et al., 2021; Umemura et al., 2017). Furthermore, emphasizing the pathological process, chronic hyperglycemia promotes microglial activation and neuroinflammation, processes that exacerbate neurodegeneration and are associated with synaptic loss at the expense of brain plasticity, reinforcing the biological plausibility of the link between DR and cognitive impairment (Kan et al., 2025; Lv et al., 2022).

The analysis of additional clinical variables revealed that arterial hypertension and multimorbidity are key determinants in the progression of cognitive decline (Newby and Garfield, 2022; Calvin et al., 2022). Chronic hypertension induces vascular remodeling, impaired cerebral autoregulation, and silent microinfarcts (Faraci, 2021), whereas multimorbidity increases systemic inflammation and disrupts metabolic homeostasis, thereby amplifying neuronal vulnerability (Zazzara et al., 2025). The lack of significant associations with dyslipidemia, depression, or overall cardiovascular risk may reflect the modulatory effect of adequate therapeutic control or the comparatively lesser influence of these factors relative to the cumulative vascular burden and multimorbidity (Zazzara et al., 2025; Kivipelto et al., 2022; Khawagi et al., 2024).

These findings underscore the importance of a comprehensive approach in the clinical management of T2DM, which goes beyond glycemic control to include prevention of cognitive decline through optimization of vascular health and effective management of comorbid conditions (Zazzara et al., 2025; Kivipelto et al., 2022).

From a clinical and translational perspective, identifying diabetic retinopathy (DR) as a marker of cognitive risk offers opportunities for early interventions and personalized medicine strategies (Cheng et al., 2021; Wu et al., 2022). The integration of regular ophthalmological and cognitive assessments would enable the early detection of individuals at risk, facilitating the implementation of prevention programs aimed at preserving cognitive function and delaying progression to dementia (Huang et al., 2024). Rigorous blood pressure control, optimization of multimorbidity, and intervention on vascular risk factors could act synergistically to mitigate neurodegeneration associated with T2DM (Mousavi et al., 2025; Launer, 2019; Patel and Edison, 2024). These multidisciplinary strategies highlight the need for coordinated approaches across specialties such as endocrinology, neurology, and internal medicine, with an emphasis on secondary prevention of cognitive decline (Bain et al., 2019; Andersen et al., 2023).

From a research standpoint, these findings point to the necessity of longer-term longitudinal studies incorporating advanced structural and functional neuroimaging techniques, as well as quantification of biomarkers related to inflammation, oxidative stress, and endothelial dysfunction (Peng et al., 2024; Yu et al., 2025). The integration of clinical, ophthalmologic, and neuroimaging data could enable the development of robust predictive models through artificial intelligence, facilitating early identification of high-risk subgroups and the application of personalized preventive or neuroprotective interventions (Wang et al., 2025; Pedersen et al., 2023; Oktem et al., 2025).

Furthermore, the exploration of pharmacological and non-pharmacological interventions aimed at improving cerebral perfusion and retinal vascular health could open new therapeutic avenues to slow cognitive decline progression in patients with T2DM (Yu et al., 2025; Pedersen et al., 2023).

In summary, DR emerges not only as a significant ocular complication but also as a clinical indicator of neurological vulnerability, given that the retina and brain share an embryological origin, the retina develops as an extension of the diencephalon and contains specialized neurons involved in early stages of visual processing. Its assessment and longitudinal monitoring could be integrated into comprehensive management protocols for T2DM (Li et al., 2024). The combined approach of glycemic control, comorbidity management, ophthalmological evaluation, and cognitive monitoring constitutes a promising strategy to reduce the burden of diabetes-associated cognitive impairment. This approach bridges endocrinology, geriatrics, and translational neuroscience, providing a framework for future research integrating biomarkers, neuroimaging, and individualized risk prediction (Wang et al., 2024; Moran et al., 2023; Rodriguez et al., 2020; Crosby-Nwaobi et al., 2013; Zhu et al., 2024).

Finally, the present findings should be interpreted in light of certain limitations. Although the longitudinal design and the use of data derived from a real-world diabetic retinopathy screening program strengthen the clinical relevance of this study, diabetic retinopathy was recorded only as a binary variable (presence/absence), and information on disease severity or advanced retinal imaging biomarkers (e.g., OCT/OCTA-derived parameters) was not available. In addition, cognitive performance was analyzed as a continuous outcome to preserve the full variability of MMSE scores and maximize statistical efficiency in the longitudinal analyses; therefore, differences according to clinically meaningful cognitive categories could not be explored. Consequently, a more detailed characterization of retinal alterations and their relationship with cognitive decline could not be fully characterized. Nevertheless, the use of routine digital fundus photography, a non-invasive, widely available examination already integrated into primary care diabetic retinopathy screening programs, enhances the potential translation of these findings into routine clinical practice. Future longitudinal studies incorporating standardized diabetic retinopathy severity grading together with quantitative retinal imaging biomarkers are warranted to further refine risk stratification and improve our understanding of the relationship between retinal and cerebral neurodegeneration.

## 5 Conclusion

In conclusion, DR may represent not only an ocular complication of T2DM but also a clinical indicator of neurological vulnerability. In this cohort study, the presence of DR was associated with a greater degree of cognitive decline at 12 months, suggesting a potential link between microvascular damage and cognitive deterioration in this population. Indeed, the presence of certain comorbidities was related to significantly lower MMSE scores across all assessments, reflecting the cumulative impact of chronic diseases on cognitive function. Similarly, hypertension showed a delayed association with poorer cognitive performance, while diabetes duration was inversely correlated with cognitive scores from the beginning of follow-up.

Furthermore, significant differences were observed in this cohort of older adults with T2DM regarding sex and sociodemographic characteristics, including clinical profiles. Specifically, women were older, had lower educational levels, and a higher frequency of widowhood, whereas men exhibited higher rates of marriage and university education, with no differences in socioeconomic status. Clinically, hypertension was the most prevalent comorbidity, followed by dyslipidemia, particularly in men, and depression with a higher frequency overall.

Taken together, these findings highlight the importance of a comprehensive approach that addresses metabolic, vascular, and psychosocial factors to preserve cognitive function and improve quality of life in this population. Moreover, incorporating cognitive assessment into the clinical follow-up of people with diabetes is essential, considering sex differences and comorbidity burden in the design of preventive strategies and personalized interventions.

## Data Availability

The raw data supporting the conclusions of this article will be made available by the authors, without undue reservation.
